# Supplementary material for: Matrix Tablets Based on Chitosan–Carrageenan Polyelectrolyte Complex: Unique Matrices for Drug Targeting in the Intestine
Source: Pharmaceuticals (Basel). 2022 Aug 9;15(8):980. doi: 10.3390/ph15080980 (PMC9412913; doi:10.3390/ph15080980)
Supplement: Supplementary file 1 [file pharmaceuticals-15-00980-s001.zip › Figure S1.pdf]

**Supplementary online material**

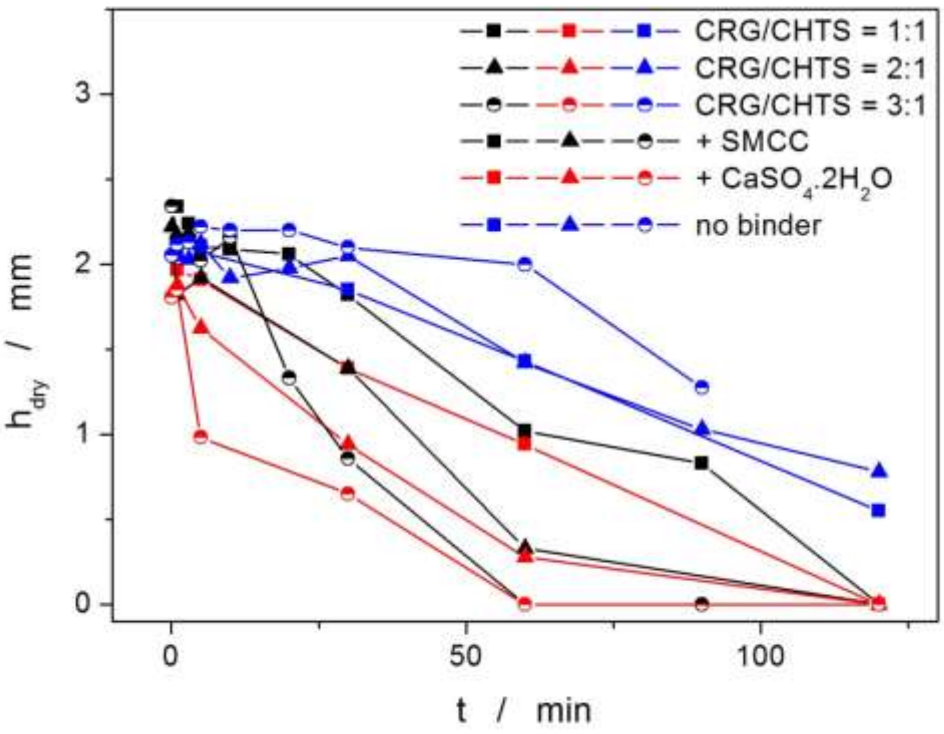


Figure S1: Evolution of the $h_{dry}$ quantity (height/thickness of the dry tablet core) during the dissolution in the acidic medium.
