# Supplementary figures and images for: Matrix Tablets Based on Chitosan–Carrageenan Polyelectrolyte Complex: Unique Matrices for Drug Targeting in the Intestine
Source: Pharmaceuticals (Basel). 2022 Aug 9;15(8):980. doi: 10.3390/ph15080980 (PMC9412913; doi:10.3390/ph15080980)

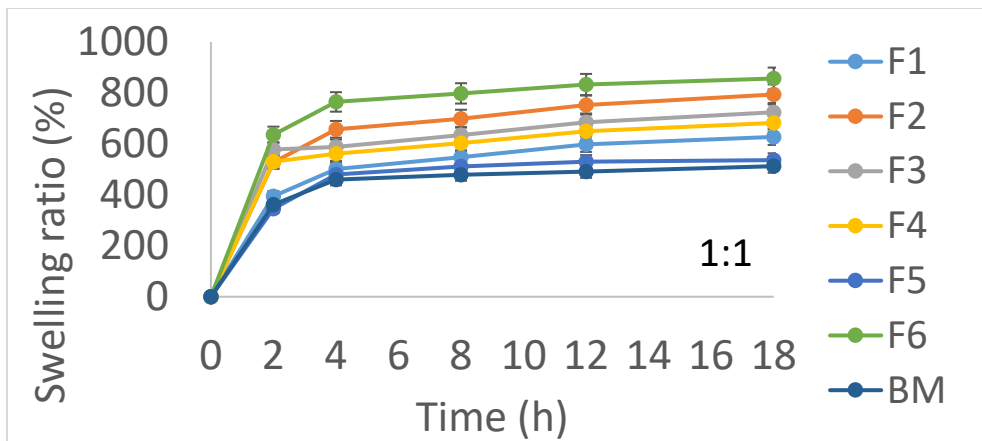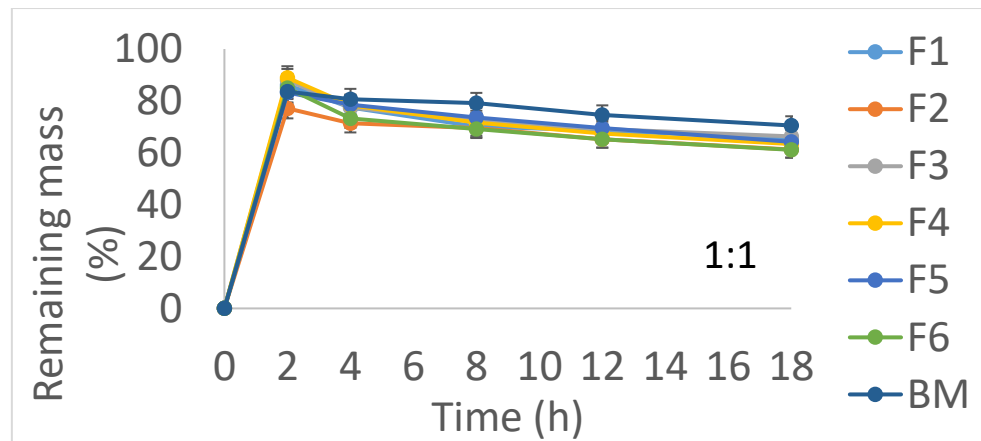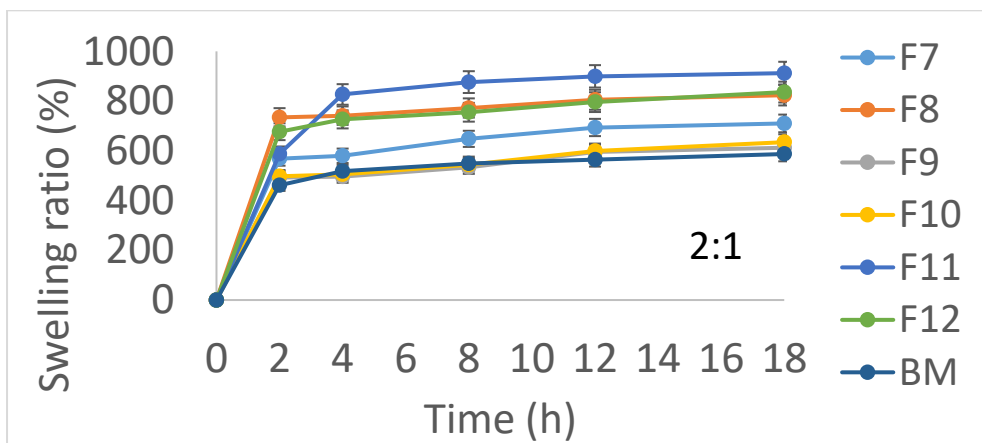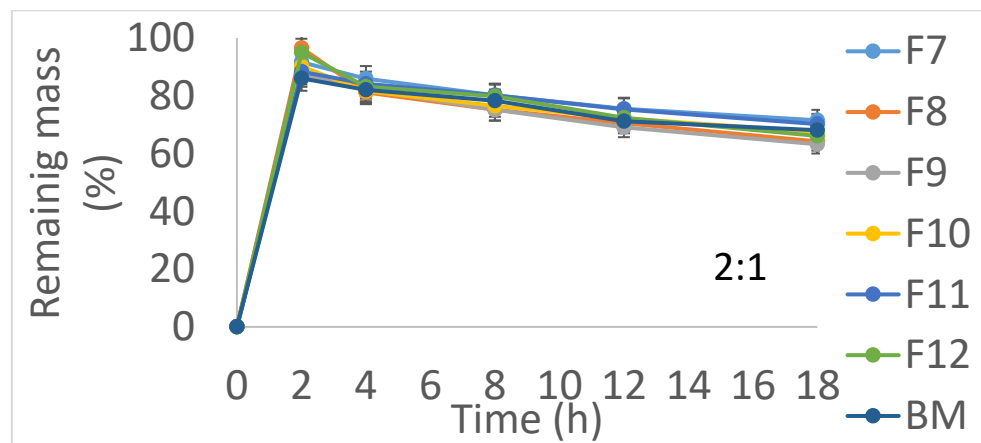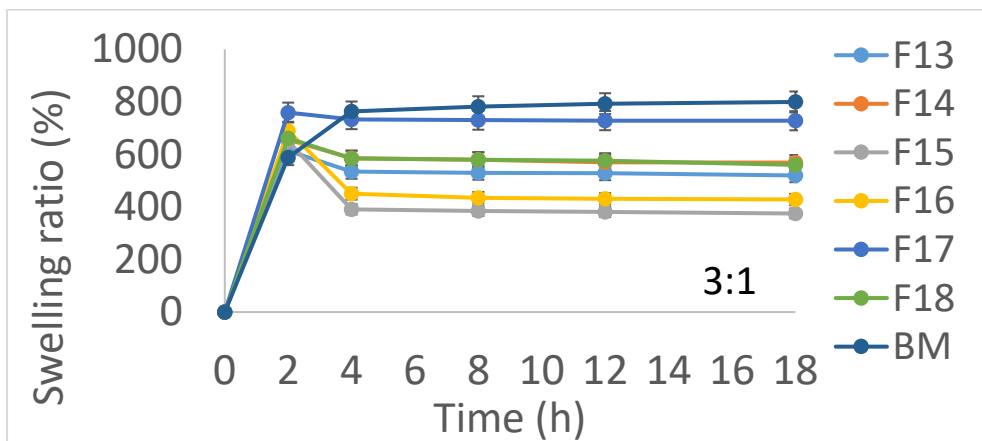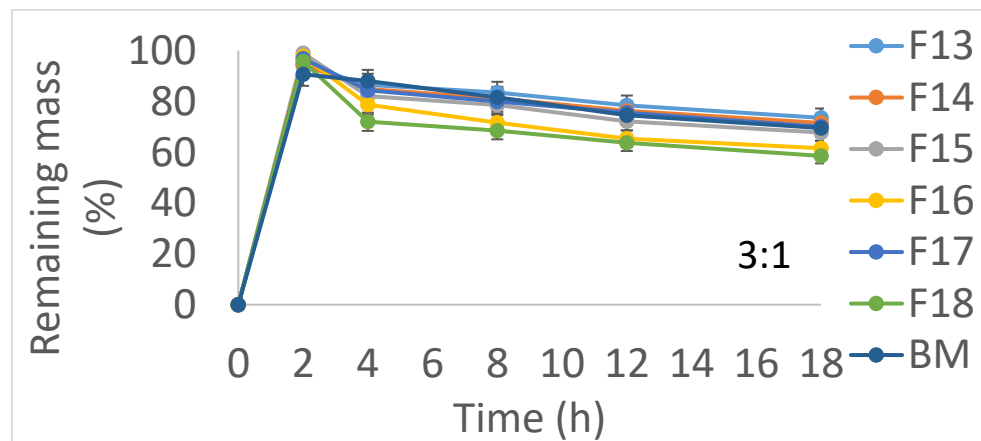

Supplement: Supplementary file 1 [file pharmaceuticals-15-00980-s001.zip › Figure S2.pdf]

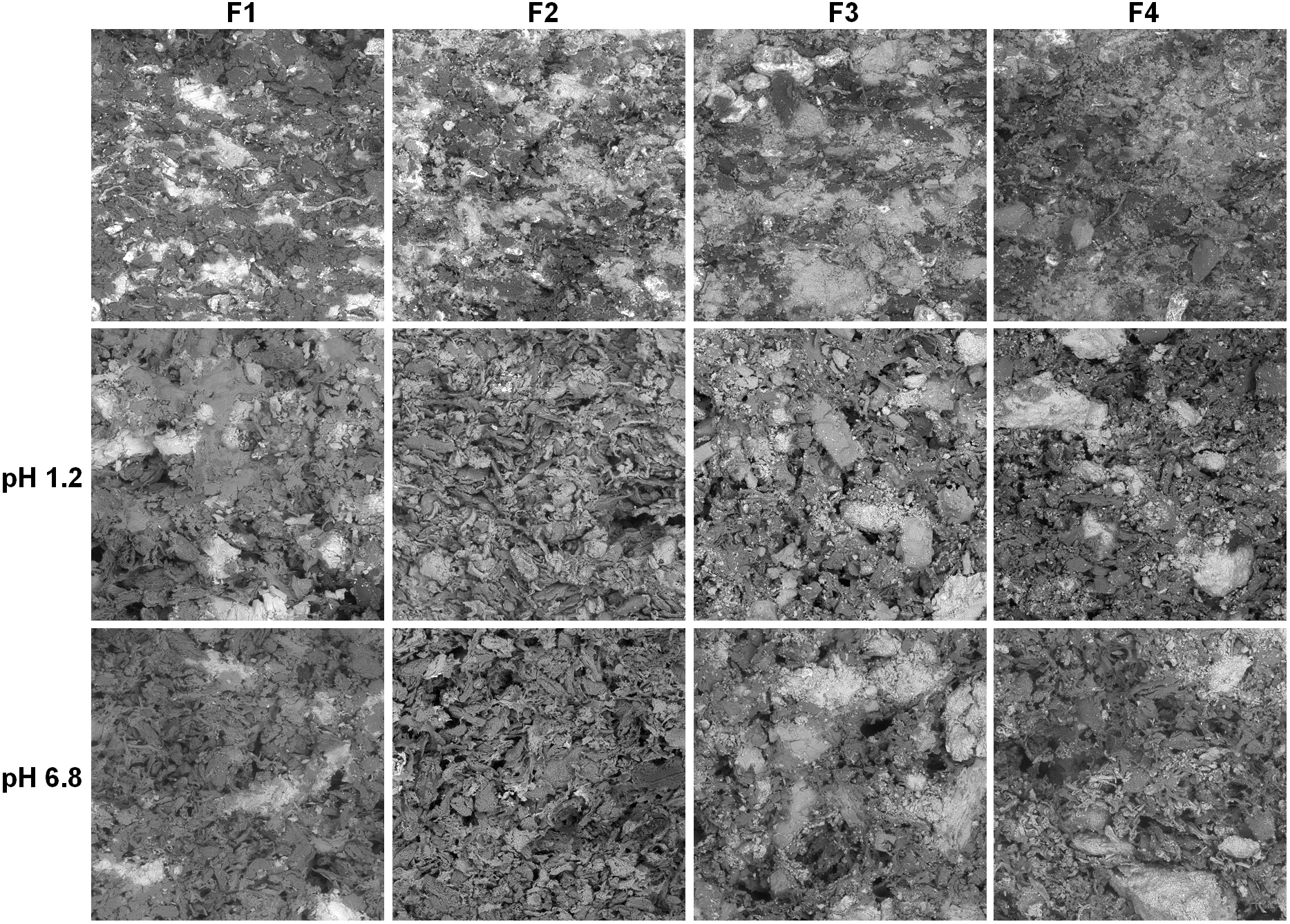

Supplement: Supplementary file 1 [file pharmaceuticals-15-00980-s001.zip › Figure S3.TIF]

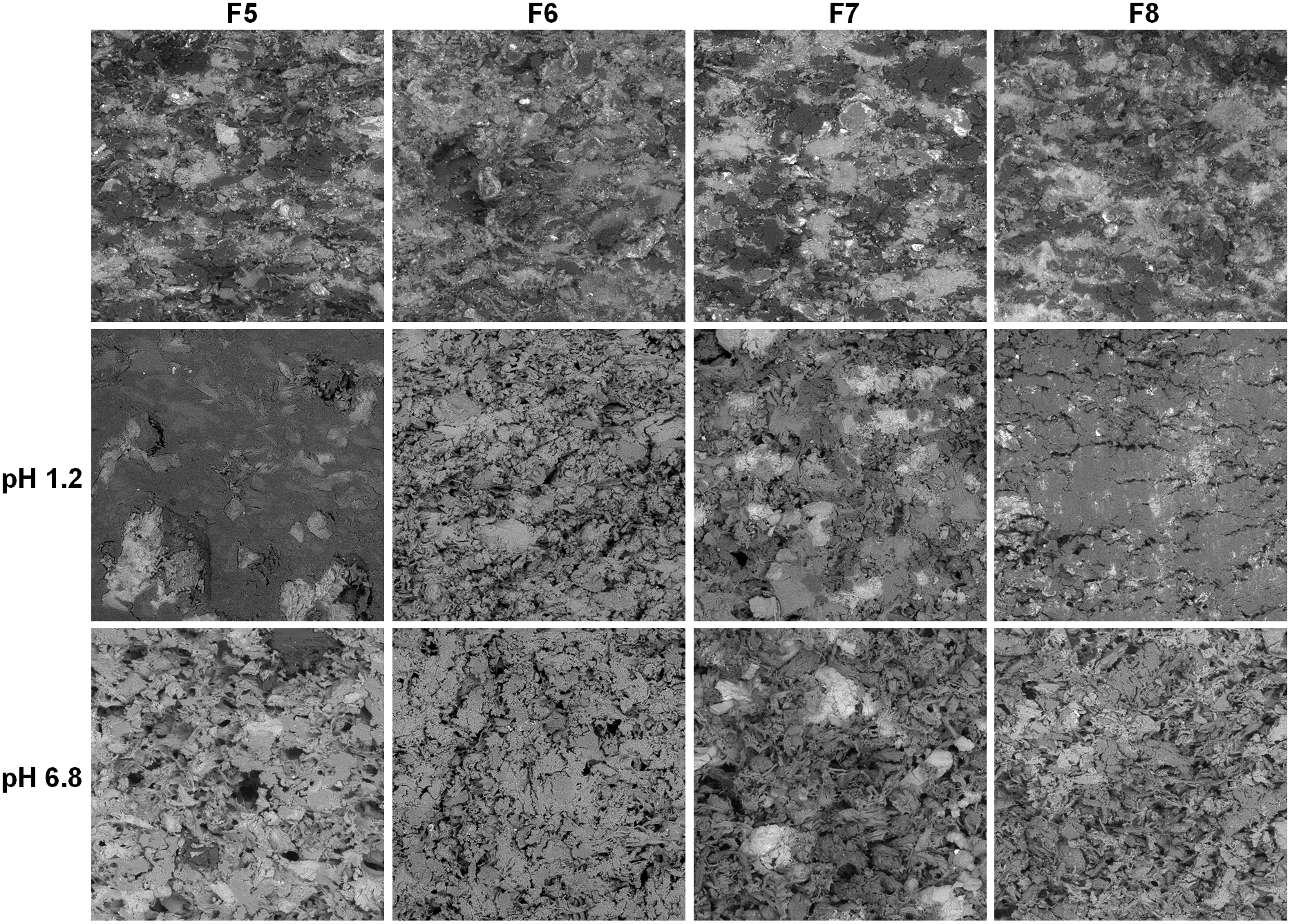

Supplement: Supplementary file 1 [file pharmaceuticals-15-00980-s001.zip › Figure S4.TIF]

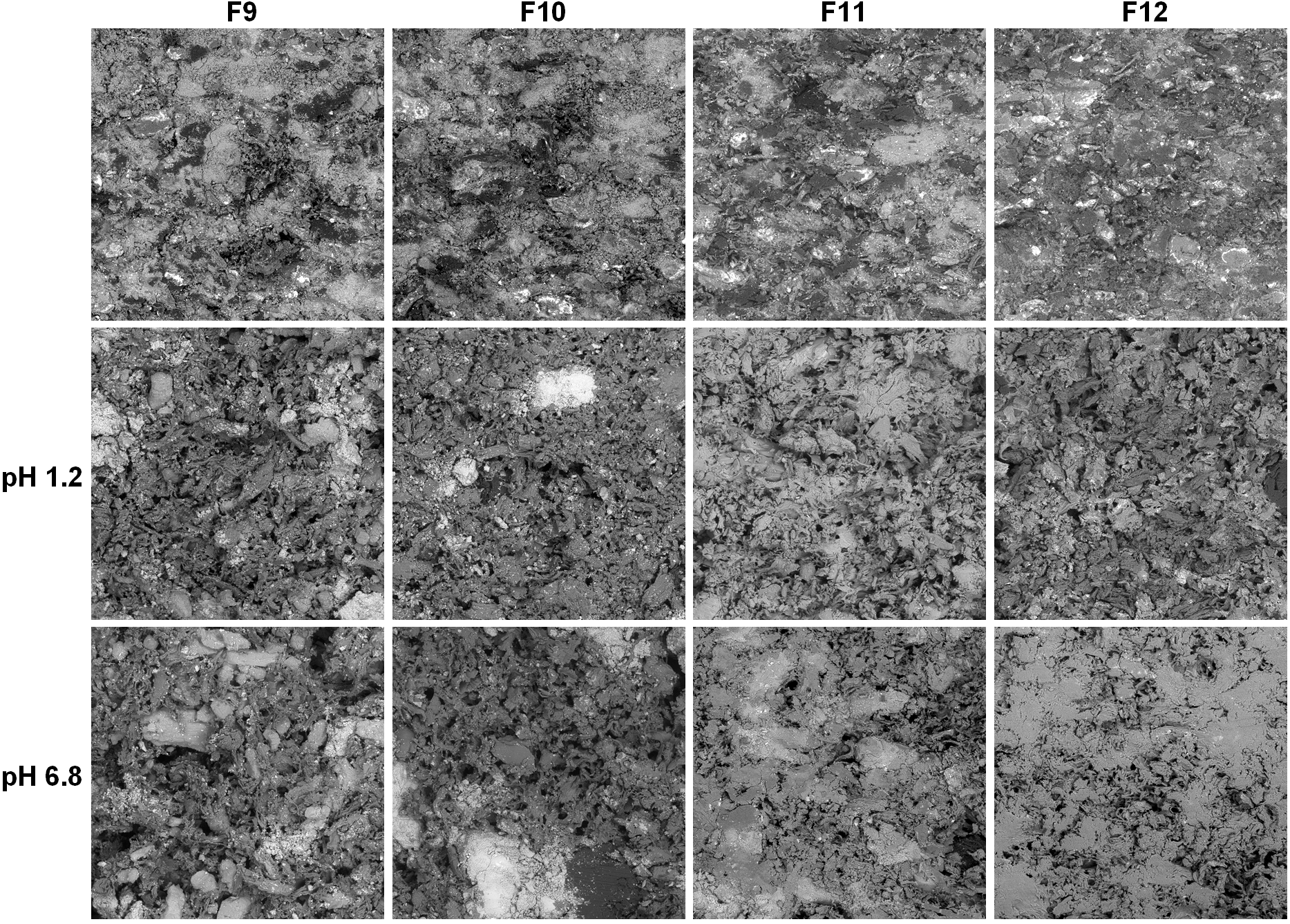

Supplement: Supplementary file 1 [file pharmaceuticals-15-00980-s001.zip › Figure S5.TIF]

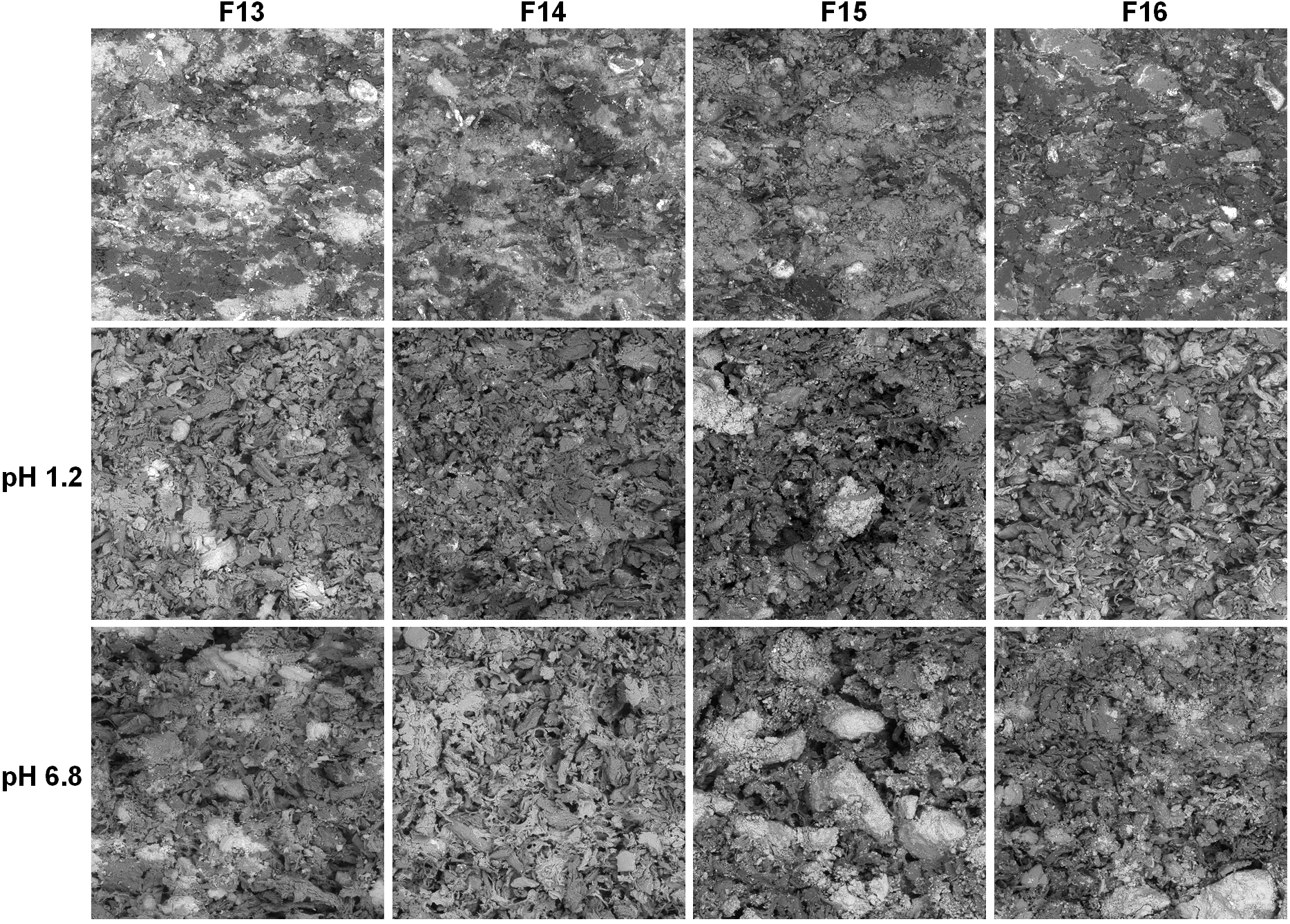

Supplement: Supplementary file 1 [file pharmaceuticals-15-00980-s001.zip › Figure S6.TIF]
